# Enhanced Nanotwinned Copper Bonding through Epoxy-Induced Copper Surface Modification

**DOI:** 10.3390/nano14090771

**Published:** 2024-04-27

**Authors:** Tsan-Feng Lu, Pei-Wen Wang, Yuan-Fu Cheng, Yu-Ting Yen, YewChung Sermon Wu

**Affiliations:** Department of Materials Science and Engineering, National Yang Ming Chiao Tung University, Hsinchu 30010, Taiwan; s0881513.c@nycu.edu.tw (T.-F.L.); nycu3116.en11@nycu.edu.tw (P.-W.W.); weddie6231.11@nycu.edu.tw (Y.-F.C.);

**Keywords:** Cu–Cu direct bonding, surface modification, grain refinement, abnormal grain growth, nanotwinned Cu

## Abstract

For decades, Moore’s Law has neared its limits, posing significant challenges to further scaling it down. A promising avenue for extending Moore’s Law lies in three-dimensional integrated circuits (3D ICs), wherein multiple interconnected device layers are vertically bonded using Cu–Cu bonding. The primary bonding mechanism involves Cu solid diffusion bonding. However, the atomic diffusion rate is notably low at temperatures below 300 °C, maintaining a clear and distinct weak bonding interface, which, in turn, gives rise to reliability issues. In this study, a new method of surface modification using epoxy resin to form fine grains on a nanotwinned Cu film was proposed. When bonded at 250 °C, the interfacial grains grew significantly into both sides of the Cu film. When bonded at 300 °C, the interfacial grains extended extensively, eventually eliminating the original bonding interface.

## 1. Introduction

For decades, Moore’s Law has approached its limits, making it extremely challenging to scale down further. One of the promising candidates for extending Moore’s Law is three-dimensional integrated circuits (3D ICs), in which several interconnected device layers are vertically bonded together using Cu–Cu bonding. They offer advantages such as lower power consumption, a smaller form factor, higher performance, and greater functional density [1,2,3]. To facilitate a rapid and effective study of the Cu–Cu bonding interface, thin-film structures have been widely employed in research, contributing significantly to the field of 3D ICs [4,5,6]. Therefore, we used Cu thin films for an initial feasibility study.

Copper stands as the predominant interconnecting material in advanced packaging technologies, playing a vital role in advanced electronic packaging technologies [7,8,9]. The primary bonding mechanism involves Cu solid diffusion bonding; however, the atomic diffusion rate is considerably low at lower temperatures, posing a drawback in terms of requiring a higher bonding temperature. Ideally, temperatures below 300 °C are preferred because elevated temperatures may lead to the warping of Si wafers due to the mismatch in thermal expansion coefficients among Si, Cu, and dielectric materials, resulting in poor coplanarity [10]. However, at bonding temperatures below 300 °C, the grain growth in the Cu joints is minimal, thus preserving the clear, distinct interface of the original bonded surface [11,12,13,14,15]. This can lead to reliability issues. Lim et al. [16] found that when the original bonding interface was eliminated, the shear strength of Cu joints could increase by 77%. Thus, eliminating the bonding interface is crucial for enhancing the electrical conductivity and mechanical strength of Cu joints.

To eliminate the bonding interface, substantial grain growth is preferred. Chen et al. [17,18,19,20,21] discovered a new type of abnormal grain growth in stable bamboo-like Cu film structures, eliminating the bonding interface. Sakai Taiji et al. [22] utilized a cutting process to create a fine-grain region on the surface of the Cu bump, which could undergo recrystallization at 150 °C, achieving the elimination of bonding interfaces at low temperatures.

Fine grains can also be produced by plastic deformation, but this method has mostly been used to generate fine grains in bulk materials [23,24,25,26], with there being very limited examples of its use in thin films. This study pioneers a new approach for inducing fine grain formation on the surface of Cu films through deformation. Specifically, the technique involves epoxy-induced deformation, leading to the establishment of a bilayer structure with a fine-grained surface. Utilizing the unique properties of these fine grains, bonding interfaces could be effectively eliminated during the Cu bonding process.

## 2. Experimental

### 2.1. Cu Film Electrodeposition

An electroplated Cu film on Si wafer was used in this study. The Si substate consisted of a 200 nm SiO_2_ layer, a 100 nm thick TiW adhesion layer, and a 200 nm thick (111)-oriented Cu seed layer sputtered by Oerlikon Cluster Line 300. In order to remove organic contaminations, the samples were cleaned with acetone and isopropyl alcohol and then immersed in citric acid solution for a few seconds to remove oxides from surface. For the anode in electroplating, a Cu sheet with a purity of 99.99% was employed. The electroplating bath consisted of a high-purity CuSO_4_ solution containing 0.8 M Cu cations and 40 ppm hydrochloric acid [19].

To electroplate the high (111)-oriented Cu films, referred to as nanotwinned Cu (Nt-Cu), 4 mL/L of a commercial additive (108C, supplied by Chemleaders Inc., Hsinchu County, Taiwan) was introduced into the electrolyte. The electroplating bath was stirred using a magnetic bar at a rate of 1200 rpm. A current density of 120 mA/cm^2^ was applied, and the deposition of Cu thin films took place at a temperature of 26 °C. The total Cu thickness was about 6 μm. After the electroplating process, the samples were diced into 2 × 2 cm^2^ pieces, and the surface of the Cu film was flattened using a chemical mechanical polishing (CMP) process.

### 2.2. Epoxy Surface Modification

Prior to the epoxy surface treatment, the samples underwent ultrasonic cleaning in deionized (DI) water and were subsequently dried with an N_2_ purge. Following this, epoxy (Epotek 353ND, provided by Epoxy Technologies Inc., Billerica, MA, United States) was applied to the Cu surface. This epoxy is a commonly used two-component thermosetting adhesive for connectors, typically cured with an imidazole hardener. The samples were then heated from room temperature to 120 °C and maintained at this temperature for 10 min for curing. Afterward, they were cooled back to room temperature, and this cycle was repeated 16 times. The completed samples, referred to as ENt-Cu (Epoxy-modified Nt-Cu), underwent the removal of epoxy using a diamond polishing film. Finally, the surface of the ENt-Cu film was flattened via a CMP process.

### 2.3. Bonding Process

**Figure 1:**
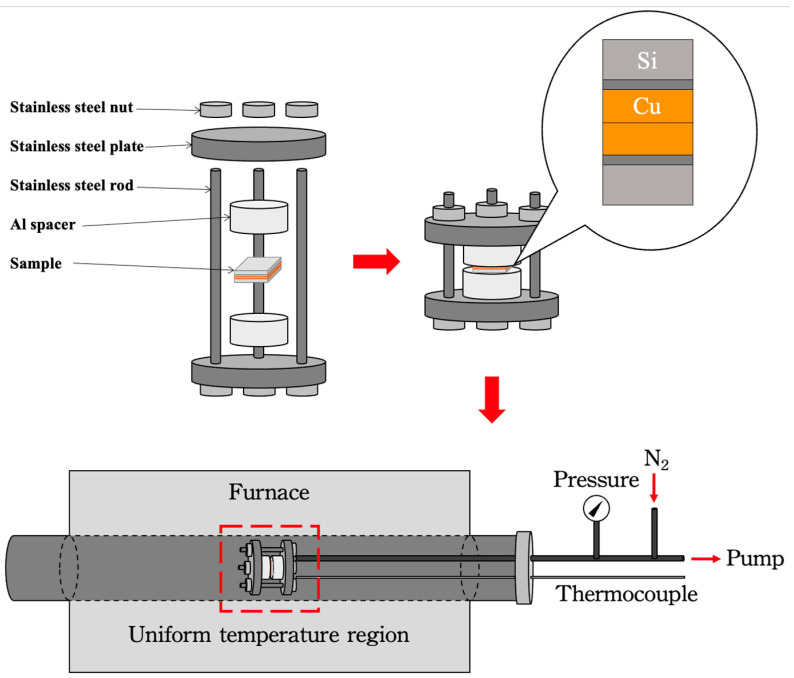
Schematic illustration of bonding processes. Note: The area within the red square represents the uniform temperature region.

Prior to bonding, the samples were diced into 1 × 1 cm^2^ pieces. They underwent ultrasonic cleaning in acetone, followed by drying with an N_2_ purge. Subsequently, the samples were immersed in a citric acid solution, rinsed with acetone and DI water, and subjected to another round of N_2_ purging. The samples were stacked in a differential thermal expansion fixture made of aluminum and stainless steel, as shown in Figure 1. The bonding process occurred at temperatures of 200 °C, 250 °C, and 300 °C, with a bonding duration of one hour under ordinary vacuum conditions (10^−3^ torr), as illustrated in Figure 1. With increasing processing temperature, the compressive stress on the sample stack also increased due to the differential thermal expansion among the various materials of the fixture. The calculated compressive stresses were 83.28 MPa at 200 °C, 96.04 MPa at 250 °C, and 117.38 MPa at 300 °C, respectively. However, determining the actual stress proved challenging as the Cu films underwent plastic deformation (creep) at elevated temperatures.

### 2.4. Material Characterizations

Prior to bonding, surface roughness was measured using atomic force microscopy (AFM, Dimension Icon Scanning Probe Microscope (ICON), Bruker, Hsinchu, Taiwan) with a 10 × 10 µm^2^ scan area. The Cu crystallographic orientation and grain size were investigated using a scanning electron microscope (SEM, JSM-7800F PRIME, JEOL, Tokyo, Japan) and a focused ion beam (FIB, Helios NanoLab 650, FEI, Hillsboro, Oregon, United States) equipped with an electron backscattered diffraction system (EBSD). The EBSD systems utilized were NordlysMax3 and Oxford Symmetry 2 for SEM and FIB, respectively. The mean grain size was determined by measuring grains in a 15 µm × 15 µm^2^ EBSD image. Transmission electron microscopy (TEM, JEOL-2100F, Tokyo, Japan) was employed to confirm the microstructure of Cu films. The strain was verified using Kernel Average Misorientation (KAM), a useful mode in EBSD.

## 3. Results and Discussion

### 3.1. Morphology and Crystallographic Orientation of Cu Films

**Figure 2:**
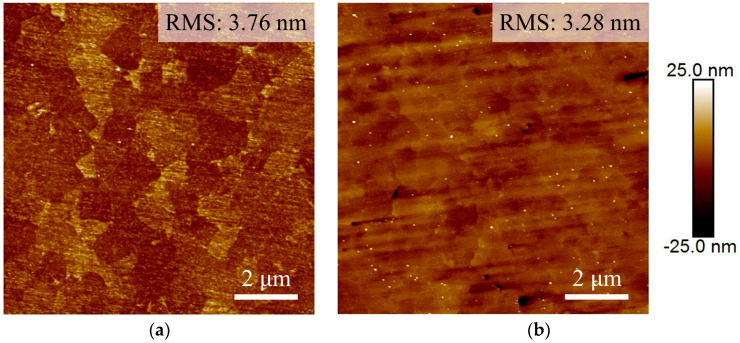
Typical AFM images of (**a**) Nt-Cu and (**b**) ENt-Cu surfaces.

The surface roughness of the Cu samples was assessed using Atomic Force Microscopy (AFM). The root mean square (RMS) value for Nt-Cu was determined to be 3.76 nm, while for ENt-Cu, the RMS value measured 3.28 nm, as shown in Figure 2. The surface roughness of the two Cu samples did not differ much after CMP.

**Figure 3:**
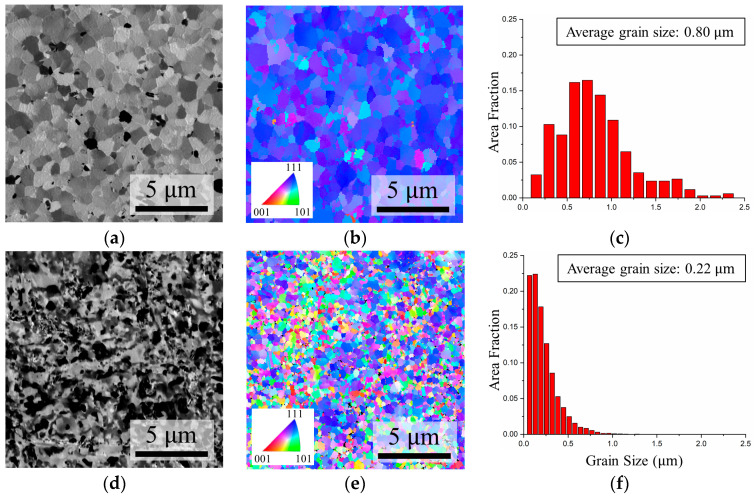
Microstructures of initial Cu films. (**a**) Plane-view SIM image of Nt-Cu. (**b**) Plane-view EBSD OIM image of Nt-Cu. (**c**) Distribution of grain sizes for the grains in (**b**). (**d**) Plane-view SIM image of ENt-Cu. (**e**) Plane-view EBSD OIM image of ENt-Cu. (**f**) Distribution of grain sizes for the grains in (**e**).

The surface morphology and crystallographic orientation of both Nt-Cu and ENt-Cu were thoroughly examined using scanning ion microscopy (SIM) and an EBSD orientation image map (OIM) to confirm their initial microstructure. Figure 3a,b show that Nt-Cu displays relatively larger grains and a pronounced preference for the (111) orientation, with over 94% of the surface area occupied by (111)-oriented grains. Figure 3c reveals that the majority of grain sizes in Nt-Cu were distributed within the range of 0.25 to 1.25 μm, with an average value of approximately 0.80 μm.

In contrast to Nt-Cu, ENt-Cu exhibited noticeable changes in surface structure and grain orientation, as shown in Figure 3d,e. Plan-view EBSD OIM images confirm the transition of the Cu surface from a highly (111)-preferred orientation to a random orientation. Additionally, the average grain size decreased from 0.80 μm to 0.22 μm. Figure 3f further illustrates that more than half of the grain sizes in ENt-Cu have dimensions below 0.25 μm.

The formation of fine grains on the surface of ENt-Cu can be attributed to two reasons: (1) the epoxy curing process and (2) the coefficient of thermal expansion (CTE) mismatch between the epoxy and the Cu during the thermal cycle process. During the epoxy curing process, Cu forms a surface oxide layer, which hydrates to produce surface hydroxyl groups under normal environmental conditions [27]. Epoxy resins are highly polar. These polar groups are the sites for the formation of strong electromagnetic bonding attraction (hydrogen bonding) between epoxy molecules and metal oxides. Finally, volume shrinkage and the restriction of molecular movement may generate stresses at the resin-Cu interface. Residual stresses also persist in the adhesive after curing, providing the impetus for storing strain energy in the Cu. However, the main reason for stress generation is the difference in CTE between epoxy and Cu [28,29,30].

Epoxy resin, with the CTE provided by Epoxy Technology, exhibits a CTE range of 54 − 206 $\times \;10^{-6}$/°C, which is approximately 10 times greater than that of Cu (17 $\times \;10^{-6}$/°C). Therefore, during cooling, the resin shrunk more than Cu, which resulted in compressive stresses being placed on the Cu film. The same occurs during heating, where the expansion of the resin caused tensile stresses on the Cu film. Both of these stresses increased the strain energy within Cu film and caused the deformation of the Cu. This introduced a large number of defects on the surface of the Cu film, causing the microstructure to change to fine grains.

**Figure 4:**
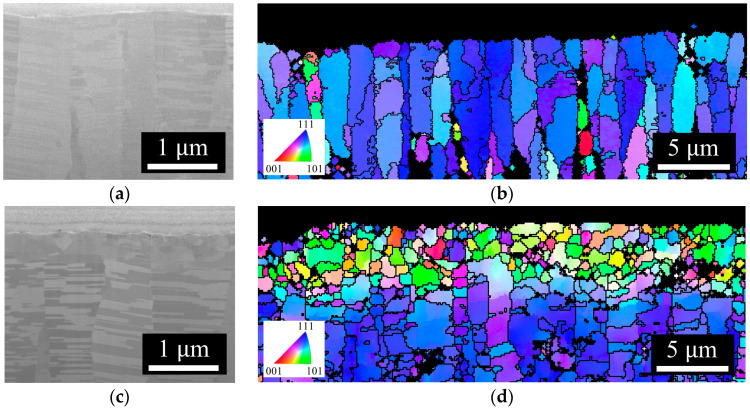
(**a**,**c**) are cross-sectional SEM images of Nt-Cu and ENt-Cu, respectively. (**b**,**d**) are cross-sectional EBSD OIM images of Nt-Cu and ENt-Cu.

The morphology of Nt-Cu and ENt-Cu was also examined through cross-sectional SEM and EBSD OIM images. Figure 4a,c show Nt-Cu films with a (111) preferred orientation. The microstructure consisted of regular columnar nanotwinned grains. In comparison with Nt-Cu, after surface modification, as shown in Figure 4b,d, the columnar nanotwinned grains under the surface transformed into fine grains with random orientations, while the entire underlying structure remained unchanged as the original nanotwinned Cu. The thickness of the fine grain layer was less than 1 µm.

**Figure 5:**
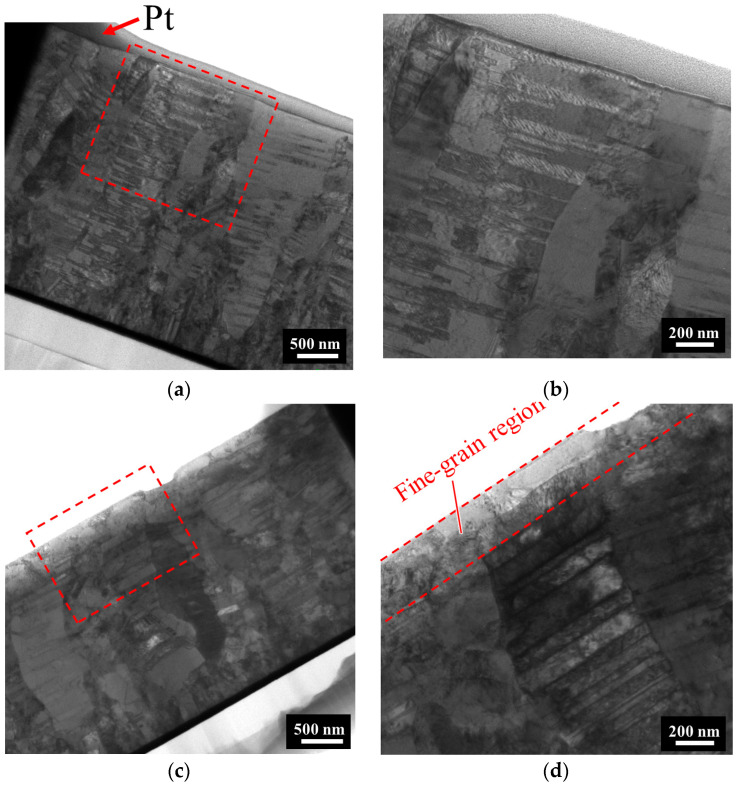
Cross-sectional TEM images: (**a**) as-fabricated Nt-Cu film; (**b**) high-magnification image of red dotted area in (**a**). (**c**) ENt-Cu film; (**d**) high-magnification image of red dotted area in (**c**). Note: the Pt layer is a standard process required for fabricating TEM samples.

The transmission electron microscopy (TEM) images provided insights into the microstructure of Nt-Cu and ENt-Cu. The nanotwinned columnar grains of Nt-Cu extending to the surface were contrasted with the region of fine grains beneath the surface in ENt-Cu. Figure 5b,d present an enlarged view of the TEM image for the specified region, offering a closer examination of the microstructural details.

It is worth noting that the atomic diffusivity of the fine-grained surface layer was greatly improved compared to the normal-grained material. This improvement was attributed to the fact that grain boundaries can serve as fast diffusion channels [5,31]. This enhanced atomic diffusivity was beneficial for the subsequent bonding process.

### 3.2. Effect of Grain Boundary Energy on Bonding Interface

**Figure 6:**
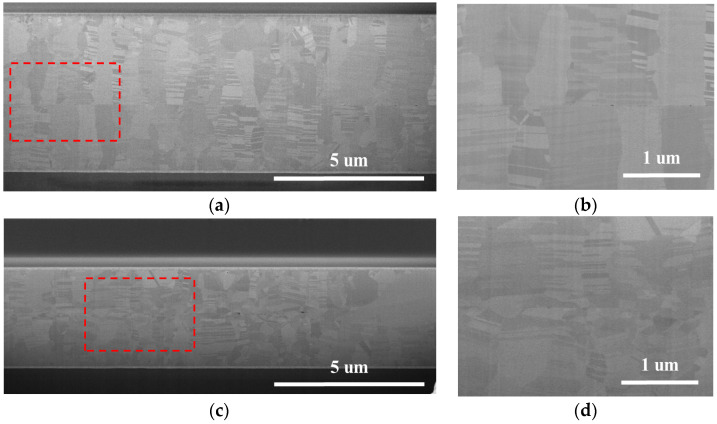
Cross-sectional SEM images after bonding at 200 °C for 1 h: (**a**) Nt-Cu; (**b**) high-magnification image of the red dotted area in (**a**). (**c**) ENt-Cu; (**d**) high-magnification image of the red dotted area in (**c**).

**Figure 7:**
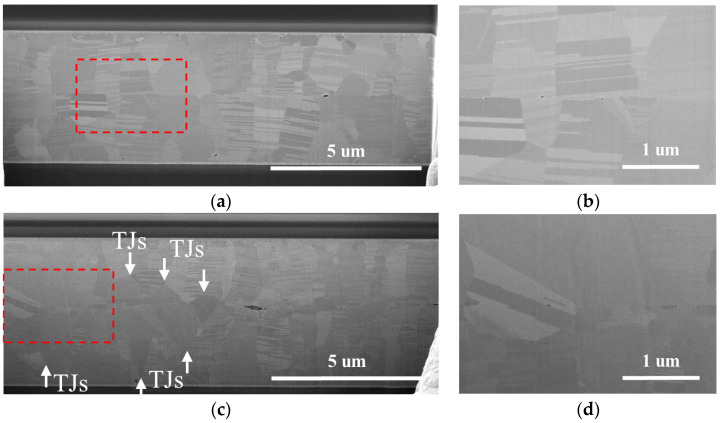
Cross-sectional SEM images after bonding at 250 °C for 1 h: (**a**) Nt-Cu; (**b**) high-magnification image of red dotted area in (**a**). (**c**) ENt-Cu; (**d**) high-magnification image of red dotted area in (**c**). Note: The white arrows indicate the locations of triple junctions.

The cross-sectional SEM images in Figure 6 and Figure 7 provide insight into the bonding interfaces (interfacial grain boundary, IGB) after the samples were bonded at 200 °C and 250 °C, respectively. The Nt-Cu sample exhibited a clear and distinct bonding interface/IGB, maintaining a microstructure almost identical to the as-deposited Nt-Cu films. The columnar grains and nanotwinned structures remained intact, with minimal grain growth across the bonding interface, highlighting the stability of the nanotwinned structure [32]. The bonding interface retained a flat plane, indicating a relatively weak bonding interface with limited diffusion between the two Cu films [12,33].

**Figure 8:**
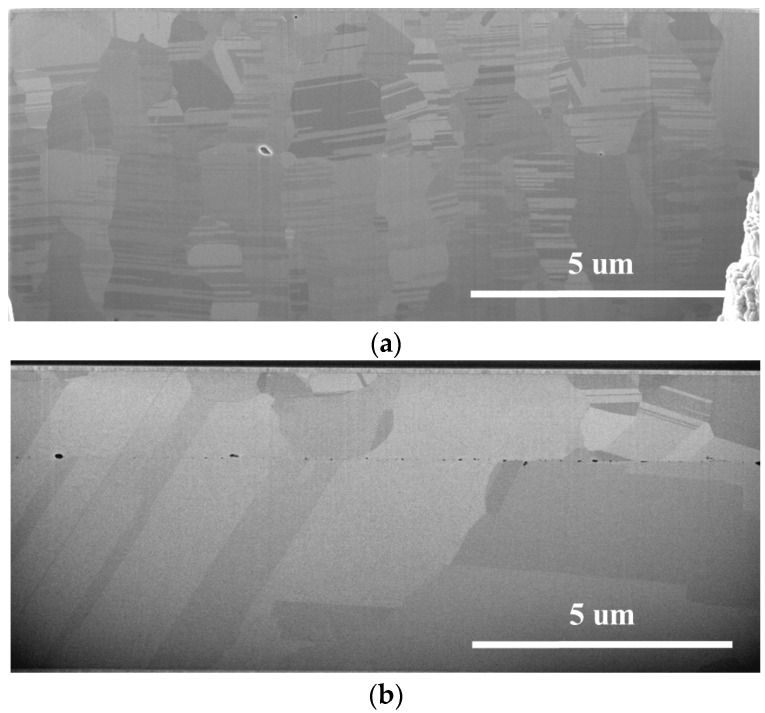
Cross-sectional SEM images after bonding at 300 °C for 1 h: (**a**) Nt-Cu; (**b**) ENt-Cu.

Upon bonding the Nt-Cu sample at 300 °C (Figure 8a), a transformation occurred in the interfacial grain boundary (IGB), transitioning from a flat plane to a zigzag plane. This alteration was attributed to the increased diffusivity of Cu at higher temperatures. Importantly, the range of the zigzag interface was observed to be smaller than that of ENt-Cu (more details on ENt-Cu will be shared later), underscoring the strong stability of the nanotwinned structure even under elevated temperature bonding conditions.

As for the bonded ENt-Cu samples, the IGB showed a zigzag shape due to grain growth and Cu interdiffusion, as shown in Figure 6 and Figure 7. The extent of these zigzag interfaces expanded with increasing bonding temperature. As shown in Figure 8b, most of the interfacial grains grew into the Cu on both sides when the bonding temperature was 300 °C. Most of the columnar grains with nanotwinned structures were consumed and transformed into large grains. In addition, the growth of interfacial grains eliminated the original bonding interface.

**Figure 9:**
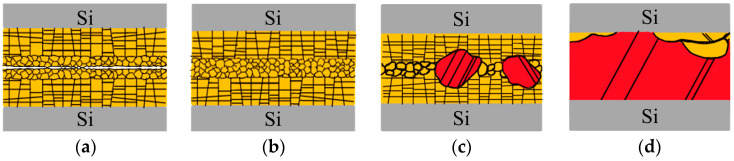
A schematic diagram illustrating the evolution of the ENt-Cu bonding interfaces bonded at (**a**) room temperature, (**b**) 200 °C, (**c**) 250 °C, and (**d**) 300 °C. As the temperature increases, abnormal grain growth occurs at the bonding interface, followed by grain growth extending into the Cu films on both sides.

Figure 9 illustrates the evolution of the ENt-Cu bonding interfaces. As shown in Figure 9a, when samples were contacted at room temperature, a significant number of voids persisted, attributed to the rough surfaces of ENt-Cu. Figure 9b depicts the bonding interface at 200 °C. In contrast to the corresponding SEM images, as shown in Figure 6c,d, most of the voids have been eliminated, and the interfacial grains have grown only slightly. Moving to Figure 9c, the interface bonded at 250 °C revealed a noticeable growth of interfacial grains, extending into the Cu films on both sides, as shown in Figure 7c,d. Figure 9d illustrates the interface bonded at 300 °C, where most of the interfacial grains extended into the Cu on both sides, and the original bonding interface was eliminated, as shown in Figure 8b.

The annealed microstructure is influenced by several factors, including surface energy, film stress, strain, twin energy, dislocation density, and grain boundaries [34]. We believe that the primary driving force behind the growth of interfacial grains in ENt-Cu is the reduction in excess energy associated with grain boundaries. A fine-grained structure is thermodynamically unstable due to the high density of grain boundaries, characterized by large boundary energy. Thus, grain growth takes place to reduce this large grain boundary energy if sufficient thermal energy is provided to the film to allow for the migration of grain boundaries [35].

Furthermore, as shown in Figure 7c, the phenomenon of grain extension into the Cu films has been extensively studied and has been shown to start from a triple junction (TJ) [36,37,38]. The driving force behind this transition was attributed to the high energy of the “T” type grain boundary junctions generated at the Cu–Cu bonding interface. In order to reduce this energy, the system tends to reconfigure triple junctions with a uniform distribution of grain boundary angles. This migration of the grain boundary results in the formation of a zigzag bonding interface [39].

### 3.3. Effect of Strain Energy on Bonding Interface

In addition to grain boundary energy, in this study, reducing the strain energy within the Cu may have also contributed to interfacial grain growth [32]. The strain energy in Nt-Cu came mainly from the high thermal stress on the Cu film due to the mismatch in CTE between Cu (17 $\times \;10^{-6}$/°C) and Si (2.5 $\times \;10^{-6}$/°C) when the temperature rose during the bonding process. In addition to the thermal stresses, the compressive stresses applied to the sample stack also increased the strain energy inside the Cu film.

As for ENt-Cu, the epoxy surface modification process imposed further stresses and strain on the Cu, in addition to those mentioned above.

**Figure 10:**
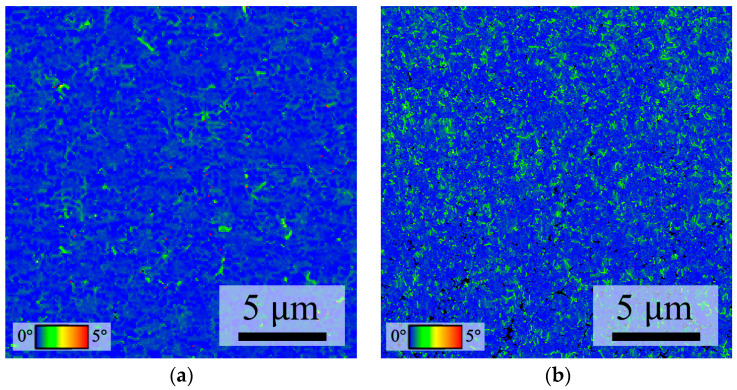
EBSD KAM maps of (**a**) Nt-Cu film and (**b**) ENt-Cu film.

The strain of the Cu film was verified using Kernel Average Misorientation (KAM), which is a useful EBSD mode for qualitatively estimating the elastic strain based on lattice misorientation [40]. Figure 10 shows KAM maps of Nt-Cu and ENt-Cu. Figure 10a shows that most of the Nt-Cu films are blue in color, representing a very low KAM degree and low dislocation density. In contrast, Figure 10b shows that the fine grains of the ENt-Cu film have a slightly higher KAM degree than Nt-Cu.

The average KAM of the ENt-Cu films was 0.32, which represents a slight increase compared to the average value of 0.23 for the Nt-Cu films. This increase in strain was attributed to the deformation during the surface modification process, which might provide additional driving forces for interfacial grains to grow and cross the bonding interface during the bonding process, thereby reducing strain energy. This growth of interfacial grains may help minimize the strain energy, grain boundary energy, and twin energy [36].

### 3.4. Effect of Epoxy Modification on Cu Films

**Figure 11:**
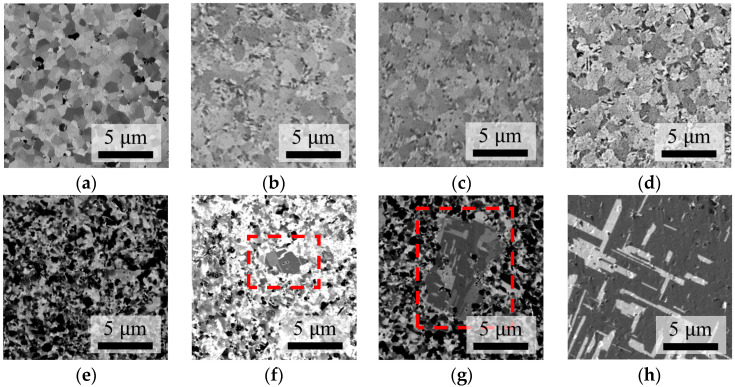
Plane-view SIM images of Nt-Cu and ENt-Cu films after annealing at various temperatures for 30 min. (**a**–**d**) Nt-Cu: (**a**) As deposited, (**b**) Annealed at 150 °C, (**c**) Annealed at 180 °C, and (**d**) Annealed at 275 °C. (**e**–**h**) ENt-Cu: (**e**) As deposited, (**f**) Annealed at 150 °C, (**g**) Annealed at 180 °C, and (**h**) Annealed at 275 °C. Note: AGG occurs in the red dotted areas in (**f**,**g**).

To observe in detail how the microstructures of the Cu films change with temperature, the Nt-Cu and ENt-Cu films were annealed at different temperatures (without compressive load). Top-view SIM images of the as-deposited Nt-Cu and after annealing at 150 °C, 180 °C, and 275 °C for 30 min are shown in Figure 11a–d. They exhibited no signs of abnormal grain growth, and the grain sizes did not change significantly. These results again demonstrate that Nt-Cu films are thermally stable.

In contrast, the as-deposited ENt-Cu surface comprised equiaxed fine grains, as shown in Figure 11e. At elevated temperatures of 150 °C and 180 °C, abnormal grain growth with twins became apparent, as shown in Figure 11f,g. As shown in Figure 11h, most of the fine grains on the surface were replaced by large grains of more than 20 μm when annealed at 275 °C. These annealing results affirmed that the epoxy surface modification process (ENt-Cu) indeed promotes grain growth.

## 4. Conclusions

An investigation of Cu–Cu bonding, forming fine grains at the top of the nanotwinned Cu (Nt-Cu) process, has led to the development of a simple and suitable process for 3D IC manufacturing. The epoxy surface modification process was employed to induce surface fine grains through deformation. In this process, epoxy resin was applied to the Cu surface. The sample was then heated from room temperature to 120 °C; this temperature was held for 10 min, followed by cooling to room temperature, and the cycle was repeated 16 times. Finally, the epoxy was removed by polishing, and the surface of the ENt-Cu (Epoxy-modified Nt-Cu) film was flattened via a CMP process. The modification process reduced the average size of the surface grains from 0.80 μm (Nt-Cu) to 0.22 μm (ENt-Cu). Subsequently, the samples underwent bonding at temperatures of 200 °C, 250 °C, and 300 °C for one hour under standard vacuum conditions (10^−3^ Torr).

For the ENt-Cu samples, at 200 °C, the bonding process resulted in the elimination of most interfacial voids, with the interfacial grains only showing minimal growth. At 250 °C, a notable expansion of interfacial grains occurred, successfully extending into the Cu film on both sides. Bonding at 300 °C led to the extension of interfacial grains into the Cu on both sides, ultimately eliminating the original bonding interface. In contrast, for the Nt-Cu samples under the same bonding conditions, the situation of the bonding interface did not change significantly. A clear and distinct bonding interface was essentially maintained.

In conclusion, our study introduces a novel approach to surface modification, resulting in the formation of a dual-layer microstructure rarely reported in Cu–Cu bonding research. Through the epoxy-induced fine grain formation process, we successfully engineered a dual-layer grain structure beneath the nanotwinned Cu (Nt-Cu) surface, characterized by significantly reduced surface grain size compared to traditional electroplated Cu. This innovative modification not only facilitates low-temperature bonding but also effectively eliminates the original bonding interface, leading to substantial improvements in bonding strength and reliability. By being able to enhance the electrical conductivity and mechanical strength of the Cu joints, our proposed epoxy surface modification process (ENt-Cu) holds promising implications for the advancement of 3D IC manufacturing technology.

## Data Availability

The data supporting the findings of this study are available from the corresponding author upon reasonable request.
